# *Paragonimus kellicotti* Flukes in Missouri, USA

**DOI:** 10.3201/eid1808.120335

**Published:** 2012-08

**Authors:** Michael A. Lane, Luis A. Marcos, Nur F. Onen, Lee M. Demertzis, Ericka V. Hayes, Samuel Z. Davila, Diana R. Nurutdinova, Thomas C. Bailey, Gary J. Weil

**Affiliations:** Washington University School of Medicine, St. Louis, Missouri, USA (M.A. Lane, L.A. Marcos, N.F. Onen, L.M. Demertzis, E.V. Hayes, S.Z. Davila, D.R. Nurutdinova, T.C. Bailey, G.J. Weil);; and John Cochran Veterans Administration Medical Center, St. Louis (D.R. Nurutdinova)

**Keywords:** paragonimiasis, Paragonimus kellicotti, flukes, crayfish, emerging pathogen, trematode, parasites, Missouri

## Abstract

You don’t have to be a contestant on Fear Factor to eat unusual things. An investigation of 9 new cases of lung fluke infection in Missouri found that in all cases, patients had eaten raw crayfish while on rafting or camping trips and most had been drinking alcohol. Although all patients recovered after treatment, a few whose diagnosis was delayed had unnecessary procedures and serious illness. Physicians should consider lung fluke infection in patients with nonspecific cough and fever, especially patients who have recently returned from a recreational river trip. Crayfish in Missouri rivers often carry lung flukes and should not be eaten raw.

## Medscape CME Activity

Medscape, LLC is pleased to provide online continuing medical education (CME) for this journal article, allowing clinicians the opportunity to earn CME credit.

This activity has been planned and implemented in accordance with the Essential Areas and policies of the Accreditation Council for Continuing Medical Education through the joint sponsorship of Medscape, LLC and Emerging Infectious Diseases. Medscape, LLC is accredited by the ACCME to provide continuing medical education for physicians.

Medscape, LLC designates this Journal-based CME activity for a maximum of 1 *AMA PRA Category 1 Credit(s)*^TM^. Physicians should claim only the credit commensurate with the extent of their participation in the activity.

All other clinicians completing this activity will be issued a certificate of participation. To participate in this journal CME activity: (1) review the learning objectives and author disclosures; (2) study the education content; (3) take the post-test with a 70% minimum passing score and complete the evaluation at www.medscape.org/journal/eid; (4) view/print certificate.

**Release date: July 20, 2012; Expiration date: July 20, 2013**

## Learning Objectives

Upon completion of this activity, participants will be able to:

- Analyze the epidemiology and microbiology of paragonimiasis
- Assess the clinical presentation of paragonimiasis
- Evaluate patterns of management of paragonimiasis
- Distinguish abnormal ancillary studies among patients with paragonimiasis

## CME Editor

**Thomas J. Gryczan, MS,** Technical Writer/Editor, *Emerging Infectious Diseases. Disclosure: Thomas J. Gryczan, MS, has disclosed no relevant financial relationships*.

## CME Author

**Charles P. Vega, MD,** Health Sciences Clinical Professor; Residency Director, Department of Family Medicine, University of California, Irvine. *Disclosure: Charles P. Vega, MD, has disclosed no relevant financial relationships.*

## Authors

*Disclosures:* ***Michael A. Lane, MD, MSc; Luis A. Marcos, MD; Nur F. Onen, MBChB, MRCP; Lee M. Demertzis, MD; Samuel Z. Davila, MD; Diana R. Nurutdinova, MD; Thomas C. Bailey, MD;*** *and* ***Gary J. Weil, MD,*** *have disclosed no relevant financial relationships.* ***Erika V. Hayes, MD,*** *has disclosed the following relevant financial relationships: received grants for clinical research from: Gilead Sciences: grant funding for ED Adolescent Rapid HIV testing project, which is now complete.*
