# Supplementary material for: Paragonimus kellicotti Flukes in Missouri, USA
Source: Emerg Infect Dis. 2012 Aug;18(8):1263–7. doi: 10.3201/eid1808.120335 (PMC3414046; doi:10.3201/eid1808.120335)
Supplement: Technical Appendix — Detailed case descriptions for 6 new patients infected with Paragonimus kellicotti flukes, Missouri, USA, September 2009–2013;October 2010. [file 12-0335_Techapp-s1.pdf]

Article DOI: http://dx.doi.org/10.3201/eid1808.120335

Paragonimus kellicotti Flukes in Missouri, USA

Technical Appendix

Patients

We report the characteristics of patients 4–9. Characteristics of patients 1–3 may be found in the report by Lane et al. (*1*).

Patient 4

Patient 1 was a 28-year-old man who sustained a traumatic eye injury during a float (recreational river) trip on the Huzzah River on August 1, 2009. He underwent surgical repair of lacerations of the upper and lower eyelids and lacrimal duct. He was treated with oral prednisone for 6 weeks. Approximately 3 weeks later, he came to a local hospital with acute right upper quadrant and right shoulder pain. This pain initially began in late June 2009. At his hospital visit, he had leukocytosis and eosinophilia. The patient had an abnormal hepatobiliary iminodiacetic acid scan result and underwent laparoscopic cholecystectomy for presumed acalculous cholecystitis. However, histopathologic analysis of the gall bladder showed normal results.

Several days after completing the course of prednisone, myalgias, nonproductive cough, and fever ≤39°C developed. He was treated by his primary care provider for influenza and community-acquired pneumonia with sequential courses of oseltamavir, azithromycin, amoxicillin/clavulanate, and linezolid without resolution of symptoms.

In October 2009, after several weeks with persistent symptoms of cough and fever, the patient came to a local emergency department. A computed tomography (CT) scan showed a moderate right-sided pleural effusion with right upper lobe consolidation and a large pericardial effusion. Laboratory studies showed a leukocyte count of 12,800 cells/mm^3 with 640 eosinophils/mm^3 (5.0%). He was transferred to our tertiary care hospital for further evaluation. At that time, he had a temperature of 38.0°C, an oxygen saturation on room air of 93%, and decreased breath sounds at the right base with dullness to percussion. Remaining physical

examination results were normal. Laboratory studies indicated a leukocyte count of 8,900 cells/mm^3 with 1,400 eosinophils/mm^3 (16%) and an erythrocyte sedimentation rate of 81 mm/h. A chest radiograph showed right upper lobe consolidation with a moderate pleural effusion.

Thoracentesis was performed and pleural fluid analysis showed orange fluid with 16,900 total cells, and 10,900 leukocytes with 66% eosinophils. The pleural fluid lactate dehydrogenase level was 2,153 IU/L and the albumin level was 3.3 mg/dL. The serum lactate dehydrogenase level was 148 IU/L and the serum protein level was 7.7 g/dL. A transthoracic echocardiogram confirmed the pericardial effusion. The patient was treated with intravenous vancomycin and cefepime. A purified protein derivative (PPD) skin test result was negative. Additional history showed that he had consumed 2 raw crayfish on a dare during a float trip on the Huzzah River in Missouri in early June 2009. His symptom onset with abdominal and shoulder pain began 3 weeks after crayfish ingestion.

A clinical diagnosis of paragonimiasis was made and antimicrobial drug therapy was discontinued. The patient was treated with oral praziquantel (25 mg/kg, 3×/d 2 days). Within 24 h, he showed had defervescence and his other symptoms improved. Sputum, fecal, and pleural fluid samples were negative for ova and parasites. Results of a *P. westermani* immunoblot performed at the Centers for Disease Control and Prevention (CDC) were negative. At follow-up 8 weeks after treatment, complete and differential blood counts were within reference ranges. A paragonimus enzyme immunoassay (Parasitic Disease Consultants, Tucker, GA, USA) showed a titer of 8, which was less than the threshold of 32 for positivity. A Western blot result with *P. kellicotti* fluke antigen performed at Washington University was positive (*2*). A repeat chest radiograph 8 weeks after treatment showed a residual, small, right-sided pleural effusion.

Patient 5

Patient 5 was a 10-year-old boy who came to his primary care physician with a 3-week history of fever, cough, and chest pain. He was treated with oseltamivir and amoxicillin for presumed influenza and bacterial superinfection. His symptoms improved, and he returned to his regular activities. However, during football practice, he sustained blunt chest trauma after which chest pain and fever returned. He was treated again with amoxicillin with no improvement, and he was referred to a local hospital for imaging.

Chest radiography showed a large, left-sided, pleural effusion and an enlarged cardiac silhouette. A chest CT scan showed large left pleural and pericardial effusions, prompting his transfer to St. Louis Children's Hospital. An echocardiogram confirmed a pericardial effusion without evidence of tamponade. Laboratory studies showed a peripheral blood eosinophil count of 1,560 cells/mm^3 (8%). Thoracentesis was performed, and examination of pleural fluid showed 3,805 cells, with 3,320 nucleated cells, 44% of which were eosinophils. No malignant cells were identified by cytologic analysis. The pleural fluid was tested by routine, fungal and mycobacterial culture, and no organisms were visualized by direct staining or on culture. He was treated empirically with cefotaxime and vancomycin but continued to have high fever despite antimicrobial drug therapy. Blood cultures and serologic testing for *Toxocara canis, Strongyloides* spp., histoplasmosis, and *Mycoplasma* spp. showed negative results. A tuberculin skin test result was negative. A repeat chest radiograph on hospital day 2 confirmed reaccumulation of his pleural effusion.

On further questioning, it was learned that the patient's uncle, an outdoor survivalist, had taught the patient to eat raw crayfish 1 year before the onset of his symptoms. Approximately 3 months before onset of his illness, the boy demonstrated his survival skills to his cousins during a family outing on the Current River in southeastern Missouri by ingesting a raw crayfish. On the basis of this history and his clinical manifestations, he was treated with praziquantel (25 mg/kg, 3×/d for 2 days) for a presumptive diagnosis of paragonimiasis. His chest pain and other symptoms promptly improved and he was discharged. At a follow-up clinic visit 1 month after treatment, his symptoms, eosinophilia, and effusions had resolved. A paragonimus immunoblot on acute-phase and convalescent-phase serum samples was performed at CDC and results were negative. However, results of a Western blot with *P. kellicotti* fluke antigen at Washington University were positive (*2*).

Patient 6

Patient 6 was a 20-year-old man with no medical history who came for medical care with a 2-week history of fever, diarrhea, and night sweats in September 2009. He was evaluated by his primary care physician who prescribed azithromycin for acute gastroenteritis. Routine stool cultures at that time were negative. Dyspnea and anorexia developed, and a 10-lb weight loss prompted return to his primary care physician in early November 2009. A chest radiograph at that time showed moderate bilateral pleural effusions.

The patient was admitted to a hospital. His absolute eosinophil count was 1,300 cells/mm^3, and a CT scan of the chest showed moderate bilateral pleural effusions and a small pericardial effusion. Transthoracic echocardiography showed a pericardial effusion with cardiac tamponade. Pericardiocentesis were performed with placement of pericardial drain Pericardial fluid had a leukocyte count of 60,800 cells/mm^3 with 50% eosinophils. Thoracentesis of a left pleural effusion yielded 1,200 mL of yellow–green fluid with a leukocyte count of 1,952 cells/mm^3 (55% eosinophils), a protein level of 6.6 g/dL (serum protein 8.0 g/dL), a lactate dehydrogenase level of 2,295 IU/L, a glucose level <5 mg/dL, and negative cytologic and routine microbiological result. After these procedures, the patient's dyspnea improved, and the pericardial drain was removed after several days. Prednisone (60 mg/day) was given empirically, and the patient was discharged shortly thereafter.

Over the next 3 months, peripheral eosinophilia and pleural effusions recurred coincident with tapering of corticosteroids, prompting 2 thoracenteses, both of which showed lymphocytic exudates. Corticosteroids had been discontinued in January 2009, one month before the patient's first visit to the pulmonary clinic at our institution. He reported persistent, low-grade exertional dyspnea and cough with sputum. Upon detailed questioning regarding travel history and exposures, the patient reported eating raw bratwurst during a camping trip 9 months earlier. Vital signs were normal, and physical examination showed decreased breath sounds and tactile fremitus with dullness to percussion at both lung bases. The absolute eosinophil count was 2,200 cells/mm^3, the IgE level was 259 IU/mL, stool ova and parasite examination results were negative, and a PPD skin test result was negative. Serum was sent to CDC for serologic analysis, and a chest radiograph showed a small left pleural effusion. The patient was unable to provide a sputum sample.

Several weeks later, results for a *Strongyloides* indirect hemagglutination assay performed at CDC were positive (7.29 U/mL, reference value <1.7 U/mL). Ivermectin was prescribed; 2 doses of 0.2 mg/kg were to be taken 2 weeks apart. Subsequently, a paragonimiasis immunoblot performed at the CDC showed positive results. A Western blot with *P. kellicotti* fluke antigen performed at Washington University showed positive results (*2*). Praziquantel (25 mg/kg, 3×/d 2 days) was prescribed. After discussing the test results with the patient, he disclosed that acting on a dare from the younger members of his party, he and several other

friends had eaten raw crayfish while intoxicated during the float trip on the Jacks Fork River in June 2009, nine months before the time of diagnosis and 12 weeks before the onset of symptoms.

At a follow-up visit 1 month after treatment with praziquantel, the patient was asymptomatic. The absolute eosinophil count was 900 cells/mm^3, and a chest radiograph showed a small, residual, left pleural effusion.

Patient 7

Patient 7 was a previously healthy 22-year old man who had with midepigastric pain, fever $\leq$38.3°C, subjective chills, and night sweats in August 2009. Shortness of breath, cough, and hemoptysis gradually developed, Two weeks after symptom onset, he awoke from sleep with the sudden onset of left-sided pleuritic chest pain.

He was admitted to a hospital where laboratory studies showed a leukocyte count of 8,100 cells/mm^3. The differential cell count was 66% neutrophils, 15% lymphocytes, 7% monocytes, and 10.5% eosinophils (absolute count 850 cells/mm^3). A CT of the chest showed a filling defect in the posterior basal segment of the right lower lung lobe, peribronchovascular opacities at both lung bases, a density in the right lower lobe, and a 3-mm nodule in the right upper lobe. A D-dimer test result was increased. Levels of thyroid-stimulating hormone, factor V Leiden, lupus anticoagulant, antinuclear antibody, antithrombin III, protein S, and protein C were within reference ranges. A transthoracic echocardiogram result was normal. Bilateral lower extremity venous duplex ultrasound did not show evidence of thrombi. The patient was treated with heparin and then warfarin for possible pulmonary embolism.

The patient continued to have cough, hemoptysis, night sweats and noted a 10–15-lb weight loss. In November 2009, a repeat chest radiograph showed a small pleural effusion and a pulmonary infiltrate. He was treated with azithromycin for 5 days, followed by cefuroxime for 7 days, and showed no improvement in symptoms. Repeat chest radiographs showed increasing pleural effusions. He was again admitted to the hospital and treated with moxifloxacin. A thoracentesis was performed, and 2 L of thin yellow-orange fluid was removed. The cell count was 1,112 cells/mm^3 with 32% neutrophils, 61% lymphocytes, and 3% eosinophils. Routine bacterial, acid-fast bacilli, and fungal cultures were negative. Pleural fluid cytologic analysis showed peripheral blood cells and mesothelial cells. Results for hepatitis were negative, and levels of antinuclear antibody, cyclic citrulline peptide, cardiolipin, β-2 glycoprotein, and

rheumatoid factor were within reference limits. A PPD skin test result was negative. The patient's course was complicated by *Clostridium difficile* infection requiring treatment with metronidazole.

In January 2010, he was hospitalized because of increasing shortness of breath and continued hemoptysis, cough, and night sweats. Imaging showed a recurrence of the right pleural effusion and opacities in the left mid and lower lungs. He underwent a video-assisted thorascopic decortication of the right lung, and 1.5 L of murky fluid was drained. Cultures were not performed and cell counts were not performed for the fluid.

The patient continued to have night sweats, productive cough with rusty-colored sputum, and progressive weight loss (20 lbs). His fever and chills had resolved. Over the course of the next 4 months, progressive shortness of breath and chest pain developed. Laboratory studies showed a leukocyte count of 11,400 cells/mm^3 with an absolute eosinophil count of 800 cells/mm^3 (7.4%). Test results for double-stranded DNA and Sjögrens syndrome antinuclear SSA and SSB antibodies were within reference limits. Repeat chest radiograph showed bilateral pleural effusions. A chest CT scan showed a 10 mm–thick pericardial effusion but no other evidence of pulmonary embolism; enlarged mediastinal and epicardial lymph nodes and a moderate pleural effusion were noted. Corticosteroid therapy was initiated for treatment of a possible connective tissue disorder.

In June 2010, the patient's mother read a newspaper article about recent cases of paragonimiasis and brought her son to our clinic for further evaluation. At that time, the patient reported that he had eaten a raw crayfish on a float trip on the Jack's Fork River in southern Missouri in July 2009, ≈4–6 weeks before onset of his illness. His physical examination was notable for dullness to percussion at the right base. Paragonimus ova were seen on examination of sputum. Results of a paragonimus immunoblot performed at CDC were positive. A Western blot result with *P. kellicotti* fluke antigen at Washington University was positive (*2*). The patient was treated with praziquantel, 25 mg/kg, 3×/d for 3 days. At a follow-up visit ≈4 weeks after completing therapy, the patient's cough, hemoptysis, and night sweats had resolved. Dyspnea had improved, but had not resolved. Eosinophilia had resolved (absolute eosinophil count 200 cells/mm^3). The chest radiograph showed bilateral, small-to-moderate pleural effusions. He had no residual symptoms 6 months after treatment.

Patient 8

Patient 8 was a healthy 30-year-old man who in July 2010 had a 2-week history of fever $\leq$38.9°C, night sweats, right-sided chest pain, productive cough with brown sputum and watery diarrhea. He was seen by his primary care physician and given a diagnosis of community-acquired pneumonia after a chest radiograph showed a right lung infiltrate and right-sided pleural effusion. He was treated with levofloxacin and corticosteroids for 10 days. The cough and dyspnea improved after treatment with prednisone. However, when the prednisone was discontinued, dyspnea returned and the patient noted a 10-lb weight loss.

He returned to his primary care physician 1 month later. A repeat chest radiograph showed persistent, right-sided pleural effusion. He received a second course of levofloxacin and corticosteroids, which improved his symptoms. However, symptoms again returned after stopping corticosteroids. A chest radiograph showed bilateral pleural effusions. A chest CT scan showed a left, upper-lobe infiltrate and bilateral pleural effusions. A CBC showed a leukocyte count of 15,000 cells/mm^3 with 3,100 eosinophils/mm^3 (21%).

In October 2010, the patient came to Washington University Medical Center for an outpatient evaluation. At that time, he was afebrile. Results of a physical examination were remarkable only for decreased breath sounds at the left base and some scant crackles. Laboratory studies showed a leukocyte count of 7,400 cells/mm^3 with 0.6% eosinophils. A chest radiograph showed bilateral pleural effusions. Medical history showed that the patient had consumed 2 raw crayfish from the Jack's Fork River while intoxicated 2 weeks before symptom onset. Fecal and sputum examination results for ova and parasites were negative. However, a paragonimus immunoblot performed at CDC showed a positive result. A Western blot with *P. kellicotti* fluke antigen performed at Washington University showed a positive result (*2*). The patient was treated with praziquantel (25 mg/kg, 3×/d for 2 days). At follow-up 3 weeks after treatment, the patient's symptoms had resolved.

Patient 9

Patient 9 was a 43-year-old man with hypertension and tobacco dependence who in November 2008 came to a primary care outpatient clinic in southern Missouri with a 2-month history of progressive dyspnea on exertion, chest pain, and nonproductive cough. A physical examination showed that he was afebrile, and he had decreased breath sounds bilaterally with

wheezing. Laboratory studies showed a leukocyte count of 8,900 cells/mm^3 and an absolute eosinophil count of 800 cells/mm^3 (9%). Pulmonary function tests showed obstructive disease. A chest CT showed bilateral pleural effusions. An echocardiogram showed a negative result. He was given a diagnosis of acute bronchitis and treated with moxifloxacin.

He returned for treatment September 2009 at which time he reported continued shortness of breath. A chest radiograph and chest CT scan showed a right pleural effusion. He was treated with a short course of amoxicillin, but his symptoms did not improve. In December 2009, he was referred to the John Cochran Veteran's Administration Medical Center in St. Louis for further evaluation. At this time, his leukocyte count was 7,000 cells/mm^3 with an absolute eosinophil count of 360 cells/mm^3 (5%). He underwent thoracentesis, and 1,600 mL of straw-colored fluid was drained. The fluid cell count was 952 leukocytes/mm^3 (29% neutrophils, 14% lymphocytes and 57% mesothelial cells). The patient was again treated with antimicrobial drugs but did not show clinical improvement. A CT scan 6 weeks after thoracentesis follow-up showed a persistent right lower-lobe infiltrate with reaccumulation of the right-sided pleural effusion.

In April 2010, the patient had an absolute eosinophil count of 710 cells/mm^3 (8%). In May 2010, he underwent a video-assisted thoracoscopic surgery procedure to obtain lung and pleural biopsy specimens for further evaluation of the recurrent pleural effusion. Pathologic examination of pleural tissue showed thickening of the alveolar septae and patchy chronic inflammation. No evidence of malignancy, fungal elements, or acid-fast bacilli were seen in the pleural tissue. Lung tissue did not show evidence of malignancy. Four nodular structures (diameter 2–4 mm) were observed. The lesions contained fibrous tissue with central calcification and mild chronic inflammation. Pathologic analysis of these nodules did not show evidence of malignancy or vasculitis.

Staining with Grocott methanamine silver and periodic acid–Schiff showed rare 3-mm and 8-mm yeast-like organisms without budding. Mucicarmine staining suggested the presence of capsules. Cryptococcus serum antigen, histoplasma complement fixation, and urine Histoplasma spp. antigen, blastomyces antibody and complement fixation, coccidioidomycosis complement fixation and antibody, PPD, fungal blood cultures and HIV test were all negative. Pleural and lung tissue cultures for fungi also showed negative results. The patient was treated with fluconazole in May 2010.

The patient showed no improvement in symptoms after 8 weeks of therapy with fluconazole. In addition, he had lost >20 lbs. Biopsy specimen slides were reviewed at the Armed Forces Institute of Pathology (Washington, DC, USA). Analysis did not confirm the presence of fungal elements. Additional information regarding exposures was obtained from the patient. The patient indicated that he had eaten crayfish while camping at least once a year for the past 20 years. However, he recalled consuming poorly cooked or raw crayfish while intoxicated on a camping trip ≈3 months before he initially sought care for dyspnea in 2008. A Western blot with *P. kellicotti* fluke antigen at Washington University showed a positive result (*2*). A paragonimus immunoblot performed at CDC also showed a positive result.

The patient was treated with praziquantel (25 mg/kg, 3×/d for 2 days). At the 6-month follow-up, chest radiograph showed resolution of pleural effusions. The patient had gained 25 lbs, but still had some residual shortness of breath. An immunoblot performed at CDC at the 60month follow-showed positive results. A complete blood count was within reference limits and showed no eosinophilia. At the 1-year follow-up, repeat imaging showed no recurrence of pleural effusion. The eosinophil count remained within reference limits. The patient continued to have dyspnea attributed to underlying chronic obstructive pulmonary disease.

References

1. Lane MA, Barsanti MC, Santos CA, Yeung M, Lubner SJ, Weil GJ. Human paragonimiasis in North America following ingestion of raw crayfish. Clin Infect Dis. 2009;49:e55–61. PubMed http://dx.doi.org/10.1086/605534

2. Fischer PU, Curtis KC, Marcos LA, Weil GJ. Molecular characterization of the North American lung fluke *Paragonimus kellicotti* in Missouri and its development in Mongolian gerbils. Am J Trop Med Hyg. 2011;84:1005–11. PubMed http://dx.doi.org/10.4269/ajtmh.2011.11-0027
