# Supplementary material for: Paragonimus kellicotti Flukes in Missouri, USA
Source: Emerg Infect Dis. 2012 Aug;18(8):1263–7. doi: 10.3201/eid1808.120335 (PMC3414046; doi:10.3201/eid1808.120335)
Supplement: Related texts

# Research article: *Paragonimus kellicotti* Flukes in Missouri, USA

**DOI:** 10.3201/eid1808.120335

**Authors:** Michael A. Lane, Luis A. Marcos, Nur F. Onen, Lee M. Demertzis, Ericka V. Hayes, Samuel Z. Davila, Diana R. Nurutdinova, Thomas C. Bailey, Gary J. Weil

**Affiliations:** Washington University School of Medicine, St. Louis, Missouri, USA (M.A. Lane, L.A. Marcos, N.F. Onen, L.M. Demertzis, E.V. Hayes, S.Z. Davila, D.R. Nurutdinova, T.C. Bailey, G.J. Weil);; John Cochran Veterans Administration Medical Center, St. Louis (D.R. Nurutdinova)

## Abstract

Paragonimiasis is an infection caused by lung flukes of the genus *Paragonimus*. In Asia, *P. westermani* infections are relatively common because of dietary practices. However, in North America, cases of paragonimiasis, which are caused by *P. kellicotti* flukes, are rare. Only 7 autochthonous cases of paragonimiasis were reported during 1968–2008. In 2009, we reported 3 new case-patients with paragonimiasis who had been seen at our medical center over an 18-month period. Six additional case-patients were identified in St. Louis, Missouri, USA, and treated at Washington University–affiliated health centers in 2009–2010. We report detailed descriptions of these case-patients, which includes unusual clinical manifestations. We also describe public health interventions that were undertaken to inform the general public and physicians about the disease and its mode of transmission.

Paragonimiasis is an infection caused by lung flukes of the genus *Paragonimus*. As many as 9 species of *Paragonimus* are responsible for human infections worldwide (*1*). Human paragonimiasis is common in Asia, where diets often include raw, cured, pickled, or salted crustaceans (*2**,**3*). In contrast, consumption of uncooked crustaceans is uncommon in North America.

In North America, paragonimiasis is caused by *Paragonimus* *kellicotti* flukes (*4*). *Paragonimus* spp. lung flukes have a complex life cycle, requiring snail and crustacean intermediate hosts. Definitive hosts excrete eggs in feces or sputum, which hatch in water to become ciliated miracidia. The miracidia invade soft tissue of snails where they reproduce asexually. Cercariae released from snails invade the secondary intermediate host, a crustacean. Secondary intermediate hosts for *P. kellicotti* flukes are crayfish in the genera *Cambarus* and *Orconectes*. Mammals acquire the infection when they ingest raw or undercooked crustaceans (*5*). *P. kellicotti* fluke infections have been found in cats, dogs, bobcats (*6*), raccoons (*7*), foxes (*8**,**9*), skunks (*9*), minks (*9**,**10*), and coyotes (*9*). Human infections are uncommon; only 7 cases were reported during 1968–2008 (*2**,**11**–**18*)

In 2009, we reported a cluster of 3 patients who had probable or proven paragonimiasis caused by *P. kellicotti* flukes and who were seen at a single tertiary-care center over an 18-month period (*19*). We report an additional 6 patients seen at Washington University Medical Center, St. Louis, Missouri, and at an affiliated Veterans Administration hospital over 14 months (September 2009–October 2010). The purpose of this report is to emphasize that *P. kellicotti* flukes are an emerging pathogen in Missouri, to highlight unusual clinical features observed in these patients, to educate the public in hopes of preventing new cases, and to increase awareness among the medical community to promote early diagnosis and treatment.

## Patients, Materials, and Methods

Patients with proven or probable *P. kellicotti* fluke infection seen at Washington University School of Medicine and an affiliated Veterans Administration Hospital during September 2009–October 2010 were identified at time of clinical encounter. Patient characteristics, case histories, and laboratory values were obtained from medical records by infectious disease physicians. Immunoblot tests were performed at the Centers for Disease Control and Prevention (Atlanta, GA, USA), commercial laboratories, or Washington University School of Medicine as described in the Technical Appendix.

## Results

### Clinical Features

Patient characteristics for the combined series of 9 patients are summarized in Table 1 and Table 2, and detailed case descriptions for the 6 new patients are provided in the Technical Appendix. The patients included in this series were predominantly male (88.9%), and all but 1 were adults. Patients consumed raw crayfish while on float (recreational river) trips (7/9, 77.8%), camping (1/9, 11.1%), or as a demonstration of wilderness survival skills (1/9, 11.1%). Alcohol consumption at the time of crayfish consumption was common (7/9, 77.8%). Although there were differences in timing of seeking care and signs and symptoms, patients in this series frequently had cough (100%), fever (88.9%), and eosinophilia (100%).

**Table 1:** Characteristics of 9 patients infected with *Paragonimus kellicotti* flukes, Missouri, USA, September 2009–October 2010*

Patient no.	Age, y/sex	Location	Incubation period, wk	Signs and symptoms	Time to diagnosis, wk	Method of diagnosis	Reference
1	31/M	Jacks Fork and Current Rivers	2	Fever, pharyngitis, cough, dyspnea, eosinophilia	3	Clinical history	(*19*)
2	26/F	Meramec River	2	Fatigue, cough, fever, eosinophilia	12	Serologic analysis	(*19*)
3	32/M	Current River	3	Fever, malaise, cough, headache, eosinophilia	12	Serologic analysis	(*19*)
4	28/M	Huzzah River	8	Fever, myalgia, malaise, cough, weight loss, eosinophilia	12	Clinical history	NA
5	10/M	Current River	16	Fever, myalgia, malaise, cough, chest pain, weight loss, eosinophilia	3	Clinical history	NA
6	20/M	Jacks Fork River	12	Fever, night sweats, malaise, cough, dyspnea, chest pain, weight loss, eosinophilia	36	Serologic analysis	NA
7	22/M	Jacks Fork River	6	Fever, night sweats, cough, dyspnea, chest pain, weight loss, eosinophilia	40	Serologic analysis, sputum ova and parasite examination	NA
8	30/M	Jacks Fork River	2	Fever, night sweats, malaise, cough, dyspnea, chest pain, weight loss, eosinophilia	16	Serologic analysis	NA
9	43/M	Missouri River	12	Cough, dyspnea, chest pain, weight loss, eosinophilia	83	Serologic analysis	NA

*Patients 4–9 were not previously reported. NA, not applicable.

**Table 2:** Clinical and laboratory findings for 9 patients infected with *Paragonimus kellicotti* flukes, Missouri, USA, September 2009–October 2010*

Characteristic	Value
Age, y, median (range)	28 (10–43)
Male sex	8 (88.9)
Alcohol consumption	7 (77.8)
Incubation period, wk, median (range)	4 (2–12)
Duration of symptoms before examination, wk, median (range)	2 (2–8)
Signs and symptoms	
Fever	8 (88.9)
Cough	9 (100.0)
Chest pain	6 (66.7)
Dyspnea	4 (44.4)
Night sweats	5 (55.6)
Malaise	5 (55.6)
Abdominal pain	2 (22.2)
Weight loss	7 (77.8)
Laboratory findings	
Eosinophils/mm^3^ at first examination, mean (range)	1,626 (800–3,600)
% Eosinophils at first examination, mean (range)	15 (6–30)
Positive paragonimus immunoblot result†	5 (71.4)
Positive sputum ova and parasite test result†	1 (14.2)
Radiographic findings	
Pleural effusion	9 (100.0)
Nodule	4 (44.4)
Pericardial effusion	4 (44.4)

*Values are no. (%) unless otherwise indicated. †n = 7.

Paragonimiasis can be difficult to diagnose in its early stages because of the nonspecific nature of initial symptoms. In some regions, paragonimiasis may be mistakenly diagnosed as tuberculosis. In this series of patients, initial diagnoses included pneumonia, bronchitis, influenza, gastroenteritis, acute cholecystitis, and pulmonary embolism. The median time between crayfish ingestion and the onset of clinical signs and symptoms was 4 weeks (range 2–12 weeks). The median interval between the onset of symptoms and the initial visit to health care facilities was 2 weeks (range 2–8 weeks). However, the median time from symptom onset to the correct diagnosis was 12 weeks (range 3–83 weeks). Before diagnosis of paragonimiasis, patients received multiple unnecessary medications and treatments, and these were sometimes associated with serious illness. All patients were treated with antimicrobial drugs.

*Clostridium difficile* infection developed in 1 patient after multiple courses of antimicrobial drug therapy. Six (67%) patients were treated with ≥l course of corticosteroids. One patient also underwent multiple thoracentesis procedures, and 1 of these procedures resulted in pneumothorax that required chest tube replacement. This patient also underwent decortication because of recurrent pleural effusions. One patient underwent laparoscopic cholecystectomy after having right upper quadrant pain. This finding may have been related to parasite migration across the diaphragm because the gallbladder did not show any pathologic changes.

### Laboratory Test Results

Patients with paragonimiasis often have abnormal laboratory test results that are useful for making a diagnosis. Eosinophilia has been reported in 62%–66% of patients with infection caused by *P. westermani* flukes (*20**,**21*) and in 75% of patients with paragonimiasis in North America (*19*). All patients in this series had eosinophilia at initial examination (absolute eosinophil count range 600 cells/mm^3^–2,300 cells/mm^3^, % range 5.6%–21%). Pleural fluid analysis showed eosinophilia in 3 patients. Chest radiographic findings were abnormal for all patients with paragonimiasis in North America (*19*). Pleural effusions were present in 37% of paragonimiasis patients in Asia and in 60% of previously described patients in North America (*19**,**22*). All patients in this series had pleural effusions. Other chest radiographic findings included nodules, opacities, and infiltrates. Chest computed tomography scans showed pleural thickening, pericardial thickening, pericardial effusions, and worm nodules (*23*).

Four of 6 patients in the current series had pericardial effusions documented by either computed tomography or echocardiography. Although most pericardial effusions were small and did not cause hemodynamic compromise, 1 patient had cardiac tamponade that required emergency pericardiocentesis and drain placement. Analysis of pericardial fluid showed marked eosinophilia. Eosinophilic pericardial effusions were documented in 3 children with paragonimiasis caused by *P. mexicanus* flukes in Costa Rica (*24**,**25*). Pericardial effusion has also been reported for 1 patient with paragonimiasis in Asia (*26*). Pericardial effusions have not been reported for patients with *P. kellicotti* flukes infection, although various *Paragonimus* spp. flukes have been reported to invade soft tissue (*19**,**20**,**27**,**28*) and the central nervous system (*19**,**29*).

Serologic analysis can be useful for confirming a diagnosis of paragonimiasis. However, available serologic tests have limitations. An immunoblot for *P. westermani* flukes performed at the Centers for Disease Control and Prevention (Atlanta, GA, USA) has been reported to be highly sensitive (96%) and specific (99%) (*30*). However, this assay has not been validated for *P. kellicotti* flukes. In our series, 2 patients had negative immunoblot results at the Centers for Disease Control and Prevention for samples that had been positive by Western blot with *P. kellicotti* fluke antigen at Washington University (G.J. Weil, et al., unpub. data). These patients had symptoms and abnormal laboratory test results suggestive of paragonimiasis after ingestion of raw crayfish, and their symptoms resolved after therapy with praziquantel. Diagnosis by identification of ova in sputum specimens is specific, but has low sensitivity (30%–40%) (*1*). Ova were present in sputum from only 1 patient in our series (*5*). Examination of stool for ova has low sensitivity (11%–15%) (*31**,**32*).

### Response to Therapy

Praziquantel (75 mg/kg in 3 divided doses for 2 days) is the treatment of choice for paragonimiasis in the United States (*33*). Cure rates of 71%–75%, 86%–100%, and 100% have been reported with 1-, 2-, and 3-day courses, respectively (*1**,**34*). All patients in this series were treated with praziquantel for 2–3 days, and 7 (77.8%) experienced rapid clinical improvement or cure after treatment. One patient had some residual dyspnea and chest tightness 4 weeks after treatment. These findings may have been related to the protracted time between onset of his symptoms and initiation of appropriate therapy. He was asymptomatic at the 6-month follow-up visit. One atypical patient with chronic paragonimiasis who also had preexisting chronic obstructive pulmonary disease did not notice much improvement in his chronic dyspnea after praziquantel treatment, but defervescence and a weight gain of 30 pounds represented a clear clinical response to therapy.

### Public Health Interventions

Control of this organism in the wild is not feasible because of the wide geographic distribution of crayfish and mammalian intermediate hosts that eat crayfish and serve as definitive hosts for the parasite. *P. kellicotti* flukes are highly prevalent among crayfish in rivers that are used for recreation in Missouri (*5*). Effective prevention strategies should focus on physician education to improve awareness of this disease and education targeted at the general population. We worked with public health officials to help improve awareness of this disease in physicians and in the general public. For example, we assisted the Missouri Department of Health and Senior Services in creating a health advisory (www.health.mo.gov/emergencies/ert/alertsadvisories/pdf/HAd4-30-10.pdf) for physicians in Missouri with the goal of educating physicians on the risk factors, clinical signs and symptoms, and treatment for this infection. In September 2009, we collaborated with the Missouri Department of Health and Senior Services and the Missouri Department of Natural Resources to create a warning poster (www.health.mo.gov/living/environment/fishadvisory/pdf/crayfish.pdf) that was posted at canoe rental facilities and campgrounds along rivers in Missouri. This poster warned the general public about the risk for consuming raw crayfish.

In addition, during the spring of 2010, four of the authors (M.A.L., L.M.D., T.C.B., G.J.W.) provided information to local and national print, radio, and television media to increase awareness of this infection. Three cases were identified after this media campaign. One patient sought care at our medical facility after his mother, a nurse, saw an article about paragonimiasis in her local newspaper. One patient was referred to our clinic by a friend who had seen a report on paragonimiasis on a local television station. Another patient had atypical features, but increased physician awareness helped to establish the diagnosis in this patient.

## Discussion

Although only a small number of cases of human paragonimiasis have been described in the medical literature since 1984, we have seen 9 patients with this disease in St. Louis since 2006. Five other patients with this disease in Missouri have been reported to the Missouri Department of Health and Senior Services since 2009 (P. Lo, pers. comm.). *P. kellicotti* flukes are believed to be widely distributed throughout the North America. In addition, outdoor activities such as camping and float trips when combined with alcohol consumption are not uniquely confined to Missouri. It is likely that there are case-patients in other regions who have not been given a diagnosis or treated. Although most patients reported to date have been adults, this series shows that children are also at risk for infection if they ingest uncooked crayfish.

As this patient series demonstrates, delayed diagnosis can lead to unnecessary medical treatments and procedures that can cause serious illness. Clinicians should consider the diagnosis of paragonimiasis in all patients with cough, fever, and pleural effusion with peripheral eosinophilia. We are developing a new antibody assay that may help clinicians identify and treat patients with this infection. Additional efforts to raise awareness of this parasite among physicians will potentially help appropriately identify and treat currently infected persons. These efforts should also target the general public to warn them of the dangers of consuming raw crayfish.

Technical Appendix Detailed case descriptions for 6 new patients infected with *Paragonimus kellicotti* flukes, Missouri, USA, September 2009–2013;October 2010.

## References

[1] Procop GW. North American paragonimiasis (caused by *Paragonimus kellicotti*) in the context of global paragonimiasis. Clin Microbiol Rev. 2009;22:415–46. 10.1128/CMR.00005-0819597007PMC2708389

[2] Liu Q, Wei F, Liu W, Yang S, Zhang X. Paragonimiasis: an important food-borne zoonosis in China. Trends Parasitol. 2008;24:318–23. 10.1016/j.pt.2008.03.01418514575

[3] Sharma OP. The man who loved drunken crabs. A case of pulmonary paragonimiasis. Chest. 1989;95:670–2. 10.1378/chest.95.3.6702920596

[4] Ward HB. On the presence of *Distoma westermanni* in the United States. Vet Med. 1894;1:355–7.

[5] Fischer PU, Curtis KC, Marcos LA, Weil GJ. Molecular characterization of the North American lung fluke *Paragonimus kellicotti* in Missouri and its development in Mongolian gerbils. Am J Trop Med Hyg. 2011;84:1005–11. 10.4269/ajtmh.2011.11-002721633042PMC3110363

[6] Watson TG, Nettles VF, Davidson WR. Endoparasites and selected infectious agents in bobcats (*Felis rufus*) from West Virginia and Georgia. J Wildl Dis. 1981;17:547–54.680298810.7589/0090-3558-17.4.547

[7] Cole RA, Shoop WL. Helminths of the raccoon (*Procyon lotor*) in western Kentucky. J Parasitol. 1987;73:762–8. 10.2307/32824103625429

[8] Davidson WR, Nettles VF, Hayes LE, Howerth EW, Couvillion CE. Diseases diagnosed in gray foxes (*Urocyon cinereoargenteus*) from the southeastern United States. J Wildl Dis. 1992;28:28–33.154879910.7589/0090-3558-28.1.28

[9] Ramsden RO, Presidente PJ. *Paragonimus kellicotti* infection in wild carnivores in southwestern Ontario: I. Prevalence and gross pathologic features. J Wildl Dis. 1975;11:136–41.111343310.7589/0090-3558-11.1.136

[10] Ameel DJ. More data on the lung fluke, *Paragonimus*, in North America. Science. 1931;74:493–4. 10.1126/science.74.1924.49317787398

[11] Béland JE, Boone J, Donevan RE, Mankiewicz E. Paragonimiasis (the lung fluke). Report of four cases. Am Rev Respir Dis. 1969;99:261–71.576700510.1164/arrd.1969.99.2.261

[12] Boé DM, Schwarz MI. A 31-year-old man with chronic cough and hemoptysis. Chest. 2007;132:721–6. 10.1378/chest.07-071217699148

[13] Castilla EA, Jessen R, Sheck DN, Procop GW. Cavitary mass lesion and recurrent pneumothoraces due to *Paragonimus kellicotti* infection: North American paragonimiasis. Am J Surg Pathol. 2003;27:1157–60. 10.1097/00000478-200308000-0001512883250

[14] DeFrain M, Hooker R. North American paragonimiasis: case report of a severe clinical infection. Chest. 2002;121:1368–72. 10.1378/chest.121.4.136811948081

[15] Madariaga MG, Ruma T, Theis JH. Autochthonous human paragonimiasis in North America. Wilderness Environ Med. 2007;18:203–5. 10.1580/06-WEME-CR-063R2.117896846

[16] Mariano EG, Borja SR, Vruno MJ. A human infection with *Paragonimus kellicotti* (lung fluke) in the United States. Am J Clin Pathol. 1986;86:685–7.377692410.1093/ajcp/86.5.685

[17] Pachucki CT, Levandowski RA, Brown VA, Sonnenkalb BH, Vruno MJ. American paragonimiasis treated with praziquantel. N Engl J Med. 1984;311:582–3. 10.1056/NEJM1984083031109066749230

[18] Procop GW, Marty AM, Scheck DN, Mease DR, Maw GM. North American paragonimiasis. A case report. Acta Cytol. 2000;44:75–80. 10.1159/00032623010667165

[19] Lane MA, Barsanti MC, Santos CA, Yeung M, Lubner SJ, Weil GJ. Human paragonimiasis in North America following ingestion of raw crayfish. Clin Infect Dis. 2009;49:e55–61. 10.1086/60553419681705

[20] Shim YS, Cho SY, Han YC. Pulmonary paragonimiasis: a Korean perspective. Semin Respir Med. 1991;12:35–45. 10.1055/s-2007-1006223

[21] Singh TS, Mutum SS, Razaque MA. Pulmonary paragonimiasis: clinical features, diagnosis and treatment of 39 cases in Manipur. Trans R Soc Trop Med Hyg. 1986;80:967–71. 10.1016/0035-9203(86)90275-03603646

[22] Im JG, Whang HY, Kim WS, Han MC, Shim YS, Cho SY. Pleuropulmonary paragonimiasis: radiologic findings in 71 patients. AJR Am J Roentgenol. 1992;159:39–43.160971810.2214/ajr.159.1.1609718

[23] Henry TS, Lane MA, Weil GJ, Bailey TC, Bhalla S, Chest CT. Features of North American paragonimiasis. AJR Am J Roentgenol. 2012;198. In press. 10.2214/AJR.11.753022528896PMC3832351

[24] Brenes Madrigal R, Rodriguez-Ortiz B, Vargas Solano G, Monge Ocampo Obando E, Ruiz Sotela PJ. Cerebral hemorrhagic lesions produced by *Paragonimus mexicanus*: report of three cases in Costa Rica. Am J Trop Med Hyg. 1982;31:522–6.708154610.4269/ajtmh.1982.31.522

[25] Saborio P, Lanzas R, Arrieta G, Arguedas A. *Paragonimus mexicanus* pericarditis: report of two cases and review of the literature. J Trop Med Hyg. 1995;98:316–8.7563258

[26] Yang SP, Huang CT, Cheng CS, Chiang LC. The clinical and roentgenological courses of pulmonary paragonimiasis. Dis Chest. 1959;36:494–508.1384644210.1378/chest.36.5.494

[27] Chang HT, Wang CW, Yu CF, Hsu CF, Fang JC. Paragonimiasis; a clinical study of 200 adult cases. Chin Med J. 1958;77:3–9.13573527

[28] Kagawa FT. Pulmonary paragonimiasis. Semin Respir Infect. 1997;12:149–58.9195680

[29] Shih YC. Ch’En Chang YC. Paragonimiasis of central nervous system; observations on 76 cases. Chin Med J. 1958;77:10–9.13573528

[30] Slemenda SB, Maddison SE, Jong EC, Moore DD. Diagnosis of paragonimiasis by immunoblot. Am J Trop Med Hyg. 1988;39:469–71.319569210.4269/ajtmh.1988.39.469

[31] Kim JS. A comparison of sensitivity on stool and sputum examination for diagnosis of paragonimiasis. Kiseangchunghak Chapchi. 1970;8:22–4.10.3347/kjp.1970.8.1.2212913521

[32] Shin DH, Joo CY. Prevalence of *Paragonimus westermani* in some Ulchin school children. Acta Paediatr Jpn. 1990;32:269–74. 10.1111/j.1442-200X.1990.tb00824.x2239300

[33] Drugs for parasitic infections. Med Lett. 2010;8(suppl):e1–20.628369

[34] Rim HJ, Chang YS, Lee JS, Joo KH, Suh WH, Tsuji M. Clinical evaluation of praziquantel (Embay 8440; Biltricide(R)) in the treatment of *Paragonimus westermani.* Kiseangchunghak Chapchi. 1981;19:27–37.1290271610.3347/kjp.1981.19.1.27

# Research article: *Paragonimus kellicotti* Flukes in Missouri, USA

**DOI:** 10.3201/eid1808.120335

**Authors:** Michael A. Lane, Luis A. Marcos, Nur F. Onen, Lee M. Demertzis, Ericka V. Hayes, Samuel Z. Davila, Diana R. Nurutdinova, Thomas C. Bailey, Gary J. Weil

**Affiliations:** Washington University School of Medicine, St. Louis, Missouri, USA (M.A. Lane, L.A. Marcos, N.F. Onen, L.M. Demertzis, E.V. Hayes, S.Z. Davila, D.R. Nurutdinova, T.C. Bailey, G.J. Weil);; and John Cochran Veterans Administration Medical Center, St. Louis (D.R. Nurutdinova)

## Medscape CME Quiz

## Earning CME Credit

To obtain credit, you should first read the journal article. After reading the article, you should be able to answer the following, related, multiple-choice questions. To complete the questions (with a minimum 70% passing score) and earn continuing medical education (CME) credit, please go to www.medscape.org/journal/eid. Credit cannot be obtained for tests completed on paper, although you may use the worksheet below to keep a record of your answers. You must be a registered user on Medscape.org. If you are not registered on Medscape.org, please click on the New Users: Free Registration link on the left hand side of the website to register. Only one answer is correct for each question. Once you successfully answer all post-test questions you will be able to view and/or print your certificate. For questions regarding the content of this activity, contact the accredited provider, CME@medscape.net. For technical assistance, contact CME@webmd.net. American Medical Association’s Physician’s Recognition Award (AMA PRA) credits are accepted in the US as evidence of participation in CME activities. For further information on this award, please refer to http://www.ama-assn.org/ama/pub/category/2922.html. The AMA has determined that physicians not licensed in the US who participate in this CME activity are eligible for *AMA PRA Category 1 Credits*™. Through agreements that the AMA has made with agencies in some countries, AMA PRA credit may be acceptable as evidence of participation in CME activities. If you are not licensed in the US, please complete the questions online, print the certificate and present it to your national medical association for review.

## Article Title: *Paragonimus kellicotti* Flukes in Missouri, USA

## CME Questions

**1. You are seeing a 24-year-old man with a 4-week history of cough, fever, and malaise. He was seen in an urgent care center 10 days ago and prescribed a course of macrolide antibiotics, which did not improve his symptoms. On questioning, the symptoms began after a camping trip in which the patient and his friends experimented with eating raw meat and seafood.**

**You are concerned regarding the possibility of paragonimiasis in this patient. What should you consider regarding the epidemiology and microbiology of paragonimiasis?**

A. Most cases are reported in North America

B. *P. kellicotti* requires snail and crustacean intermediate hosts

C. Humans usually are infected after eating escargot

D. *P. kellicotti* infects only humans

**2. What should you consider regarding the clinical presentation of paragonimiasis in the current case series as you evaluate this patient?**

A. All patients were female

B. Cough and fever were the most common clinical symptoms

C. Vomiting and malaise were the most common clinical symptoms

D. The onset of symptoms occurred within 48 hours of ingesting food contaminated with *P. kellicotti*

**3. What should you consider regarding the management of paragonimiasis in the current case series as you evaluate this patient?**

A. The median time from symptom onset to the correct diagnosis was 12 weeks

B. Only half of patients had received antibiotic therapy

C. No patients had received corticosteroids

D. Most patients failed to respond even to appropriate antiparasitic therapy

**4. Which of the following tests is most likely to be abnormal if this patient has paragonimiasis?**

A. Serum neutrophil counts

B. Serum eosinophil counts

C. Serum sodium levels

D. Electrocardiogram

## Activity Evaluation

**Table:** 

**1. The activity supported the learning objectives.**				
Strongly Disagree				Strongly Agree
1	2	3	4	5
**2. The material was organized clearly for learning to occur.**				
Strongly Disagree				Strongly Agree
1	2	3	4	5
**3. The content learned from this activity will impact my practice.**				
Strongly Disagree				Strongly Agree
1	2	3	4	5
**4. The activity was presented objectively and free of commercial bias.**				
Strongly Disagree				Strongly Agree
1	2	3	4	5
